# Pediatric Cancer Variant Pathogenicity Information Exchange (PeCanPIE): a cloud-based platform for curating and classifying germline variants

**DOI:** 10.1101/gr.250357.119

**Published:** 2019-09

**Authors:** Michael N. Edmonson, Aman N. Patel, Dale J. Hedges, Zhaoming Wang, Evadnie Rampersaud, Chimene A. Kesserwan, Xin Zhou, Yanling Liu, Scott Newman, Michael C. Rusch, Clay L. McLeod, Mark R. Wilkinson, Stephen V. Rice, Thierry Soussi, J. Paul Taylor, Michael Benatar, Jared B. Becksfort, Kim E. Nichols, Leslie L. Robison, James R. Downing, Jinghui Zhang

**Affiliations:** 1 Department of Computational Biology, St. Jude Children's Research Hospital, Memphis, Tennessee 38105, USA;; 2 Department of Oncology, St. Jude Children's Research Hospital, Memphis, Tennessee 38105, USA;; 3 Sorbonne Université, UPMC Univ Paris 06, F-75005 Paris, France;; 4 Department of Oncology-Pathology, Cancer Center Karolinska (CCK), Karolinska Institutet, 171 64 Stockholm, Sweden;; 5 INSERM, U1138, Équipe 11, Centre de Recherche des Cordeliers, 75006 Paris, France;; 6 Howard Hughes Medical Institute, Chevy Chase, Maryland 20815, USA;; 7 Department of Cell and Molecular Biology, St. Jude Children's Research Hospital, Memphis, Tennessee 38105, USA;; 8 Department of Neurology, University of Miami, Miami, Florida 33136, USA;; 9 Department of Epidemiology and Cancer Control, St. Jude Children's Research Hospital, Memphis, Tennessee 38105, USA;; 10 Department of Pathology, St. Jude Children's Research Hospital, Memphis, Tennessee 38105, USA

## Abstract

Variant interpretation in the era of massively parallel sequencing is challenging. Although many resources and guidelines are available to assist with this task, few integrated end-to-end tools exist. Here, we present the Pediatric Cancer Variant Pathogenicity Information Exchange (PeCanPIE), a web- and cloud-based platform for annotation, identification, and classification of variations in known or putative disease genes. Starting from a set of variants in variant call format (VCF), variants are annotated, ranked by putative pathogenicity, and presented for formal classification using a decision-support interface based on published guidelines from the American College of Medical Genetics and Genomics (ACMG). The system can accept files containing millions of variants and handle single-nucleotide variants (SNVs), simple insertions/deletions (indels), multiple-nucleotide variants (MNVs), and complex substitutions. PeCanPIE has been applied to classify variant pathogenicity in cancer predisposition genes in two large-scale investigations involving >4000 pediatric cancer patients and serves as a repository for the expert-reviewed results. PeCanPIE was originally developed for pediatric cancer but can be easily extended for use for nonpediatric cancers and noncancer genetic diseases. Although PeCanPIE's web-based interface was designed to be accessible to non-bioinformaticians, its back-end pipelines may also be run independently on the cloud, facilitating direct integration and broader adoption. PeCanPIE is publicly available and free for research use.

Massively parallel sequencing has quickly become a mainstay for genetic variation studies in many research and clinical genomics laboratories. However, the sheer abundance of data produced for a single individual means that complex and often tedious data processing and curation are required to identify potentially disease-causing mutations. The process is simultaneously burdened by the volume of novel variants, many of which have scarce information available, and the diverse, distributed nature of existing variant information resources. Variant annotation tools have been developed to assist with several aspects of this work, which can add coding and noncoding predictions and population-specific allele frequencies, as well as provide filtering options for variant prioritization (Ng et al. 2009; Wang et al. 2010; Cingolani et al. 2012; McLaren et al. 2016). Likewise, variant curation tools supporting classification for clinical pathogenicity following the American College of Medical Genetics and Genomics (ACMG) guidelines (Richards et al. 2015) have also been developed (Patel et al. 2017). Although each resource offers valuable information to help researchers classify variant pathogenicity, integrated platforms are needed to provide support for all steps of the process and to streamline analysis of the thousands to millions of variants generated by massively parallel sequencing technology. With these goals in mind, we developed the Pediatric Cancer Variant Pathogenicity Information Exchange (PeCanPIE), a cloud-based portal that provides an end-to-end workflow, beginning with a set of variants in VCF (Danecek et al. 2011) and ending with ACMG-compliant classification. PeCanPIE offers three key functions: (1) automated annotation, classification, and triage via our MedalCeremony pipeline (Zhang et al. 2015); (2) an interactive variant page and visualization tools to support expert curation and committee review; and (3) a reference database of expert-reviewed germline cancer-predisposing mutations. PeCanPIE is designed on the theme of genetic variant analysis, making it flexible for extending its application to noncancer-related genetic diseases.

## Results

### Process overview

**Figure 1.**
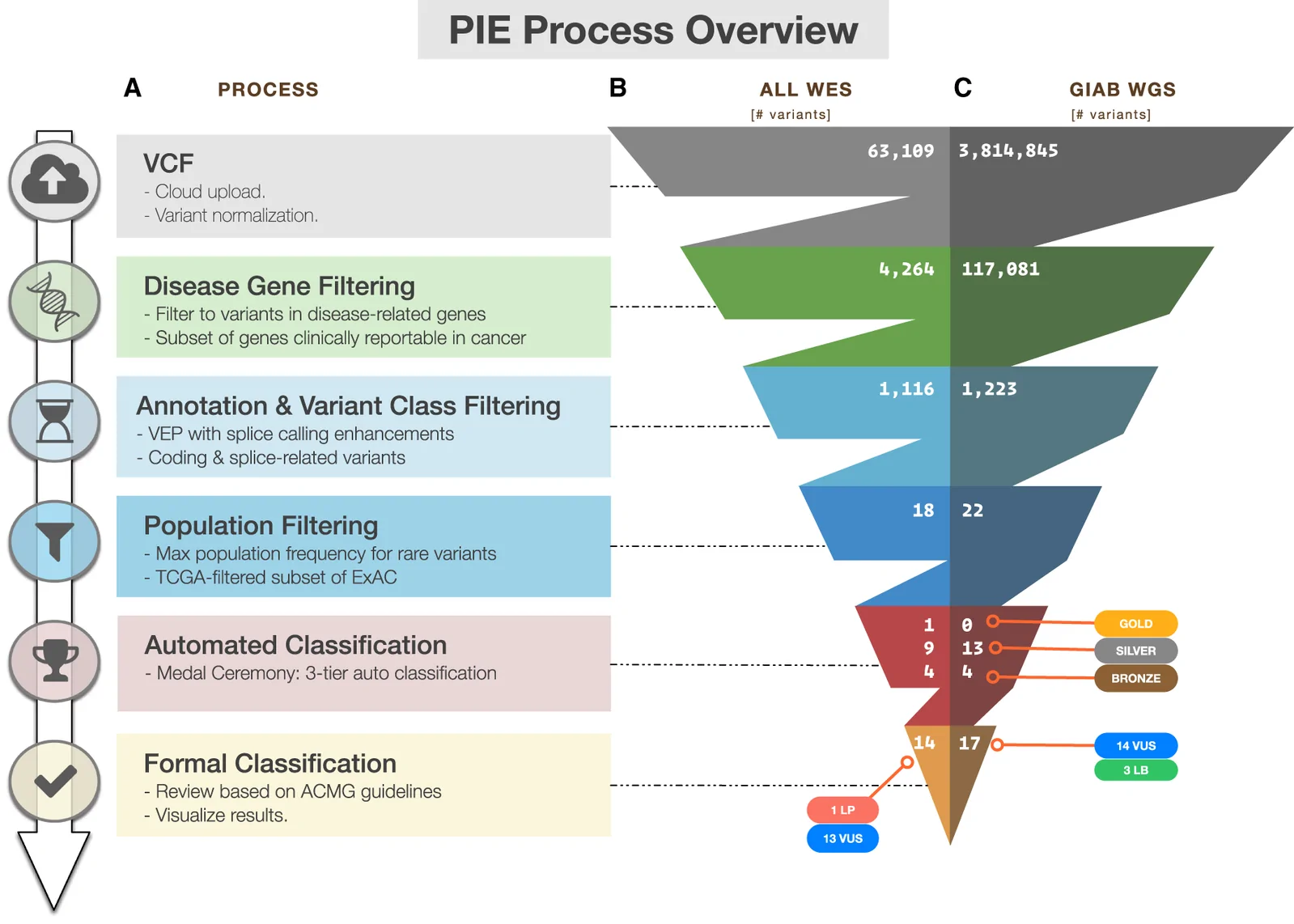
Overview of variant classification using PeCanPIE. (*A*) Overview of processing steps from VCF through ACMG-based classification. Variant counts at each processing step for whole-exome sequencing data generated from a germline sample of a patient with acute lymphoblastic leukemia (ALL), SJNORM015857_G1 (Methods) (*B*) and whole-genome sequencing data generated from Genome in a Bottle normal sample NA12878_HG001 (Methods) (*C*).

As outlined in Figure 1A, PeCanPIE launches with an interface for uploading a VCF file; alternatively, a single variant may be specified either genomically or in HGVS RNA format, which is translated into genomic coordinates via Mutalyzer (Wildeman et al. 2008). The system is native to GRCh37; GRCh38 variants are also accepted and mapped to their GRCh37 equivalent via the UCSC liftOver utility (Hinrichs et al. 2006). PeCanPIE makes use of predicted protein-coding impacts, which are expected to be identical for both reference genomes. Uploaded variants are then filtered to a set of disease-related genes (Methods; Supplemental Table S1). Users may select from predefined lists of genes from several disease categories, including cancer, cardiovascular, nonmalignant hematological, immunodeficiency, and amyotrophic lateral sclerosis (ALS) and related disorders. Alternatively, users may specify their own candidate gene list for analysis. Variants are next assigned gene and protein annotations with the Ensembl Variant Effect Predictor (VEP) pipeline (McLaren et al. 2016) and filtered by functional class and population frequency derived from the Exome Aggregation Consortium (ExAC) database (Lek et al. 2016). To ensure that pathogenic germline variants in cancer patients are retained, PeCanPIE uses the non-TCGA subset of ExAC that excludes patient samples from The Cancer Genome Atlas (TCGA) (The Cancer Genome Atlas Research Network 2008). The remaining variants are stratified into three tiers (gold, silver, and bronze) as an indication of potential pathogenicity computed by our MedalCeremony pipeline (see below). Finally, each “medaled” variant is linked to a stand-alone page featuring an interface to support semiautomated pathogenicity classification using ACMG guidelines. Two examples in Figure 1 demonstrate the classification process using VCF files generated from whole-exome sequencing (WES) of an acute lymphoblastic leukemia (ALL) patient (Fig. 1B; Moriyama et al. 2015) and whole-genome sequencing (WGS) from the Genome in a Bottle (GIAB) project (Fig. 1C; Zook et al. 2014), respectively. Only 14 of the 63,109 variants from the WES data and 17 of the approximately 4 million variants from the WGS data required expert review, which resulted in 1 and 0 pathogenic/likely pathogenic (P/LP) variants, respectively.

### Automated classification by the MedalCeremony pipeline

Automated classification of variant pathogenicity implemented in the MedalCeremony pipeline classifies variants that have a population frequency of ≤0.001 in the ExAC non-TCGA database. If desired, the frequency cutoff can be adjusted by the user or disabled altogether. Rather than the standard distribution, the non-TCGA subset of ExAC is used because as a large collection of cancer samples, TCGA is likely to be enriched for mutations related to cancer predisposition; for example, germline pathogenic mutations were previously detected in TCGA ovarian cancer samples for *BRCA1*, *BRCA*2*,* and *PALB2* (Kanchi et al. 2014). Variants appearing at a frequency higher than the cutoff may still be classified if they are present either in the International Agency for Research on Cancer (IARC) *TP53* database or in ClinVar with a classification of pathogenic or likely pathogenic and a review status of two or more gold stars (https://www.ncbi.nlm.nih.gov/clinvar/docs/review_status/). The latter exception helps retain pathogenic variants that may appear at higher population frequencies owing to founder effects, partial penetrance, or other factors impacting phenotypic heterogeneity and age at onset. To support incorporation of custom data for variant classification, users may optionally provide a “whitelist” of custom variants, matches to which will always receive a medal, or a “blacklist,” matches to which will be prevented from receiving a medal. Additional annotations are incorporated to aid with the classification process: (1) COSMIC (Forbes et al. 2008) hits; (2) functional annotations from dbNSFP (protein domain and damage-prediction algorithm calls) (Liu et al. 2013); and (3) allele frequencies in the NHLBI GO Exome Sequencing Project (ESP), the 1000 Genomes Project (The 1000 Genomes Project Consortium 2015), ExAC non-TCGA, and the Pediatric Cancer Genome Project (PCGP) (Downing et al. 2012). Although COSMIC and PCGP represent somatic rather than germline data sets, these can help inform germline classification; an example is discussed below in the “ACMG classification interface” section.

**Figure 2.**
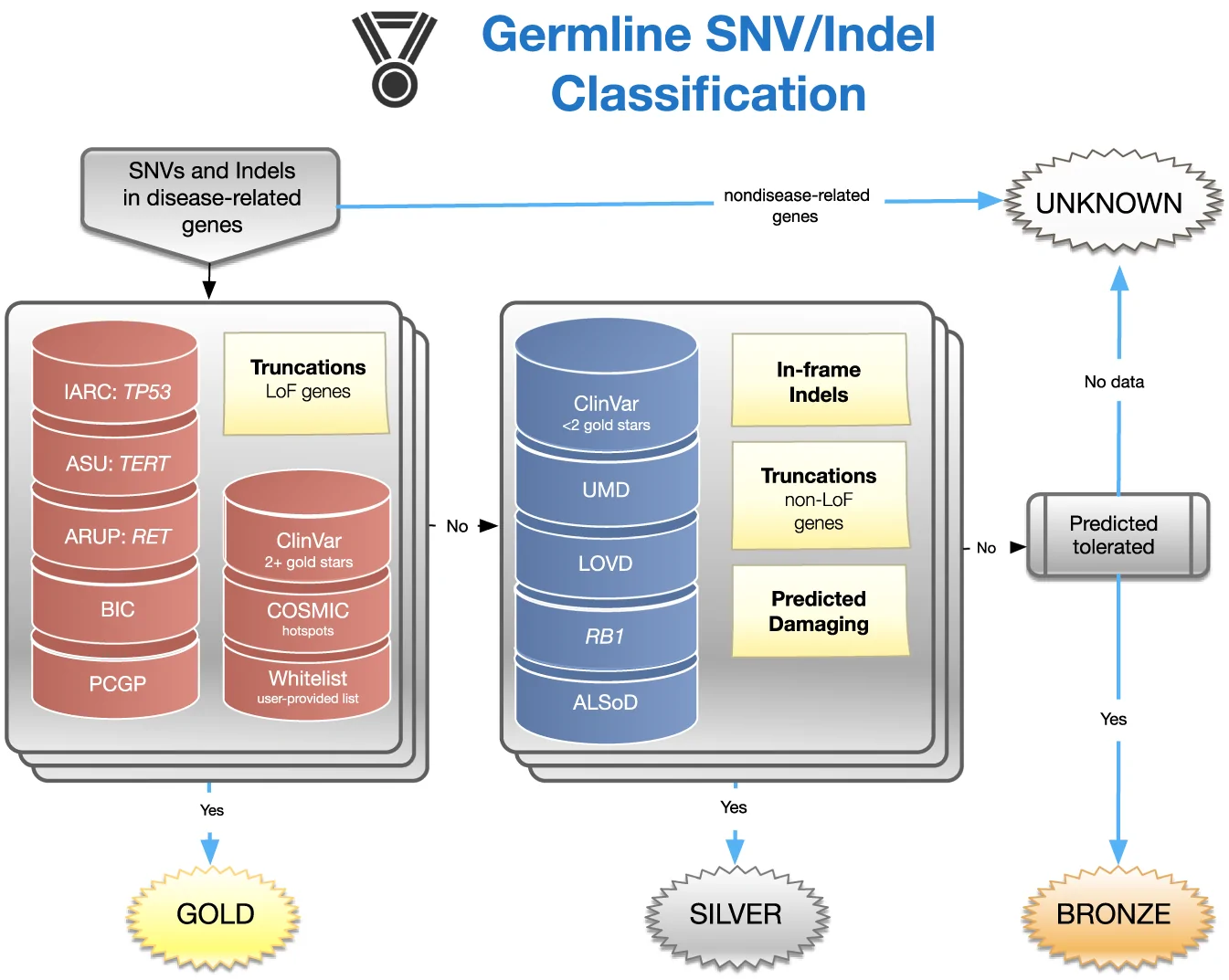
Design of the MedalCeremony pipeline for automated germline variant classification. Truncating variants in loss-of-function genes (e.g., tumor suppressors) and those matching highly curated databases receive gold medals. Truncations in non-loss-of-function genes, in-frame indels, variants predicted to be damaging, and matches to additional databases receive silver medals. Otherwise, variants predicted to be tolerated by damage-prediction algorithms receive bronze medals. Imperfect database matches receive a lower-grade medal than exact matches. Variants not meeting any of the prior criteria are labeled “unknown.”

An overview of the gold, silver, and bronze classification scheme implemented in MedalCeremony is shown in Figure 2. Gold medals are assigned to truncating variants (including splice variants) in genes where loss-of-function variants are associated with a disease (e.g., tumor suppressor genes in cancer) (Zhao et al. 2016; Chakravarty et al. 2017) as well as matches to highly curated databases, including IARC *TP53* (Bouaoun et al. 2016), ClinVar pathogenic (P) or likely pathogenic (LP) variants with a review status of two or more gold stars, Arizona State University (ASU) *TERT* (Podlevsky et al. 2007), the University of Utah Department of Pathology (ARUP) MEN2 (Margraf et al. 2009), and the National Human Genome Research Institute (NHGRI) Breast Cancer Information Core (Szabo et al. 2000). Gold medals are also assigned to somatic mutation hotspots in COSMIC (observed in ≥10 tumors after removal of hypermutators) and PCGP, St. Jude committee-reviewed germline P/LP variants, and user-provided whitelist variants, if specified. Silver medals are assigned to in-frame indels, truncation events in non–tumor suppressor genes, variants predicted to be damaging by in silico algorithms, and matches to additional databases: ClinVar P/LP with fewer than two gold stars, BRCA Share (Béroud et al. 2016), ALSoD (Abel et al. 2013), Leiden Open Variation Database (LOVD) (Fokkema et al. 2011) locus-specific databases for *APC* and *MSH2*, and *RB1* (Lohmann and Gallie 1993). Unless otherwise medaled, variants predicted to be tolerated by in silico algorithms are assigned a bronze medal. Imperfect database matches (e.g., a different allele at the same genomic position or at the same codon but with a different amino acid change) are typically assigned a lower-grade medal, for example, silver rather than gold. Variants not meeting any of the previous criteria, for example, most silent variants and those without any functional annotations, will not receive a medal. Amino acid and pathogenicity codes from the diverse variant databases used in this process are standardized to improve the reliability of annotations and utility of information (Methods). A summary of resources is shown in Table 1. MedalCeremony may also be run as a stand-alone pipeline on St. Jude Cloud (Methods).

**Table 1.**
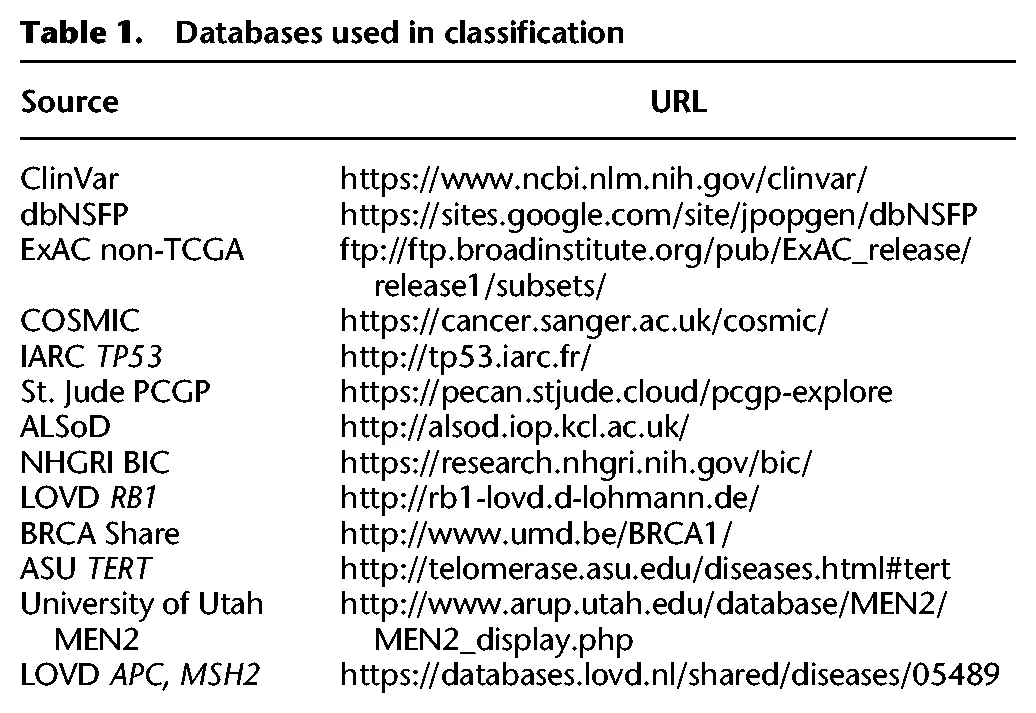
Databases used in classification

### Variant review interface

**Figure 3.**
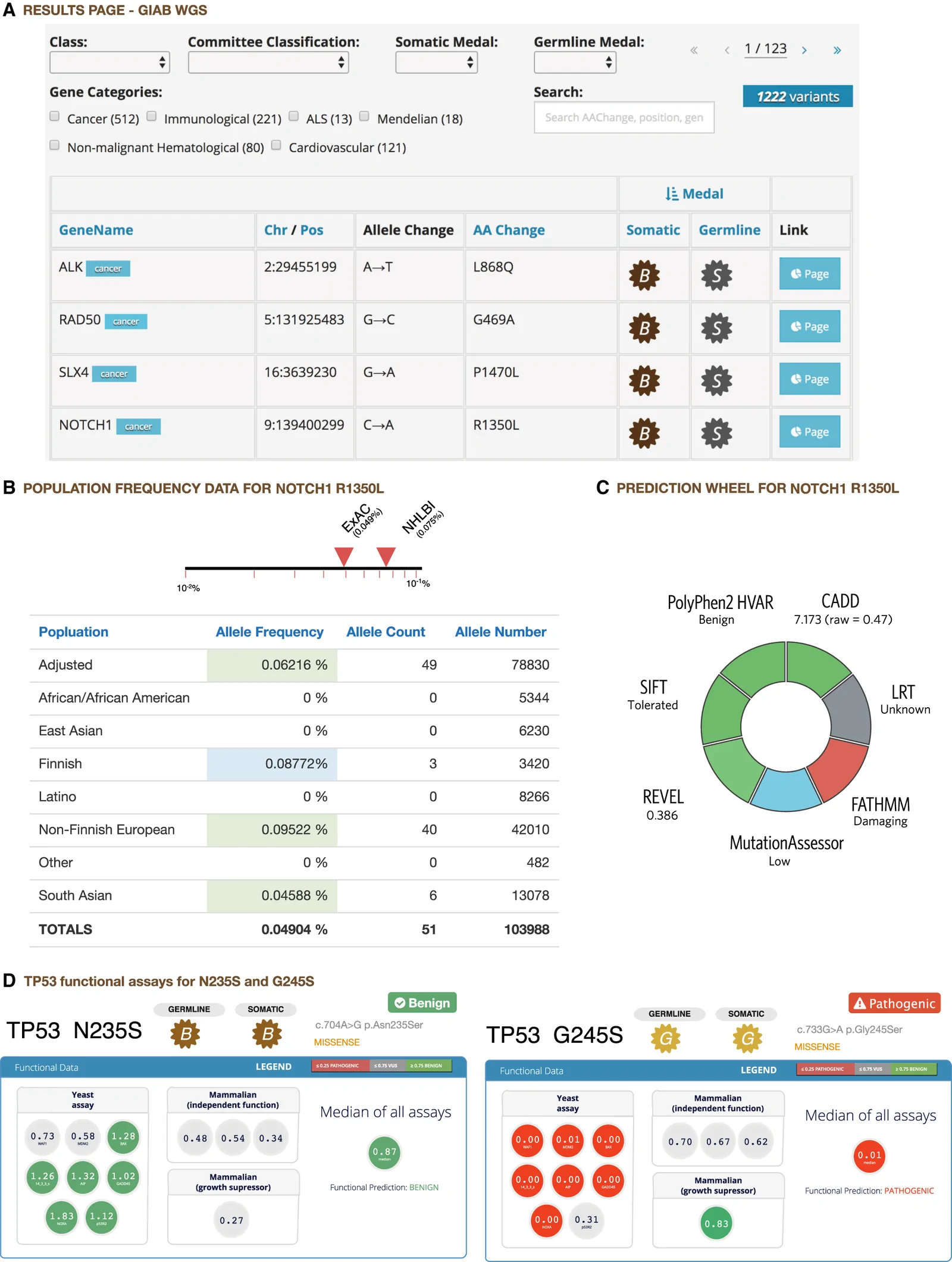
Annotation interface. Excerpts of PeCanPIE annotation interface. (*A*) Results for Genome in a Bottle WGS data set. Variant page details for NOTCH1 R1350L: (*B*) variant population frequency detail from ExAC non-TCGA database; (*C*) functional predictions. (*D*) Functional data display for *TP53* gene: Functional assay results for N235S and G245S show that N235S appears functionally benign, whereas G245S appears functionally damaging.

After MedalCeremony classification, the results are presented in a table that can be searched or filtered by gene, variant class, medal, or expert review classification (Fig. 3A). If a variant has been previously classified by the user or the St. Jude germline variant review committee, that information will be prepopulated. Variant classifications previously performed by each user are also prepopulated, allowing groups working on other diseases to establish their own expert review committees (see “Variant classification of noncancer genes” below). Each row links to a variant page containing extensive annotations, including gene information from the National Center for Biotechnology Information (NCBI) and the Online Mendelian Inheritance in Man (OMIM) database (Amberger et al. 2015), ClinVar match details, and population frequencies (Fig. 3B). Cancer predisposition genes are classified as autosomal dominant and autosomal recessive (Zhang et al. 2015) and annotated as such on the interface; this is used for determining the status of a patient but not during automated classification of individual variants. For example, in autosomal recessive genes, compound heterozygosity will be considered as having the same effect as homozygosity when a patient is signed out; this is determined when reviewing all data collected from the patient. Via dbNSFP, in silico predictions of deleteriousness are included (Fig. 3C), including REVEL (Ioannidis et al. 2016) pathogenicity scores, which fared well in a comparison of algorithms for use with ACMG clinical variant interpretation guidelines (Ghosh et al. 2017). The page also includes an embedded ProteinPaint view (Zhou et al. 2015), which overlays the current variant and other user-uploaded variants in the same data set with aggregated somatic mutations and expert-classified P/LP germline variants on the protein product. This enables visual inspection of variant recurrence, hotspots, and enrichment of loss-of-function mutations. For *TP53*, data from several variant-level databases of functional activity (Soussi et al. 2006; Giacomelli et al. 2018; Kotler et al. 2018) are presented to aid reviewers in assessing pathogenicity. Examples of pathogenic and benign variants are shown in Figure 3D; additional examples, including those classified as variant of uncertain significance (VUS) by our analysis (e.g., TP53 P222L), can be explored on our portal (https://pecan.stjude.cloud/variant/67664). In addition to *TP53*, we have also incorporated published functional data from *IKZF1* (Churchman et al. 2018) and will continue to expand the repertoire of functional data generated by basic research scientists.

### ACMG classification interface

**Figure 4.**
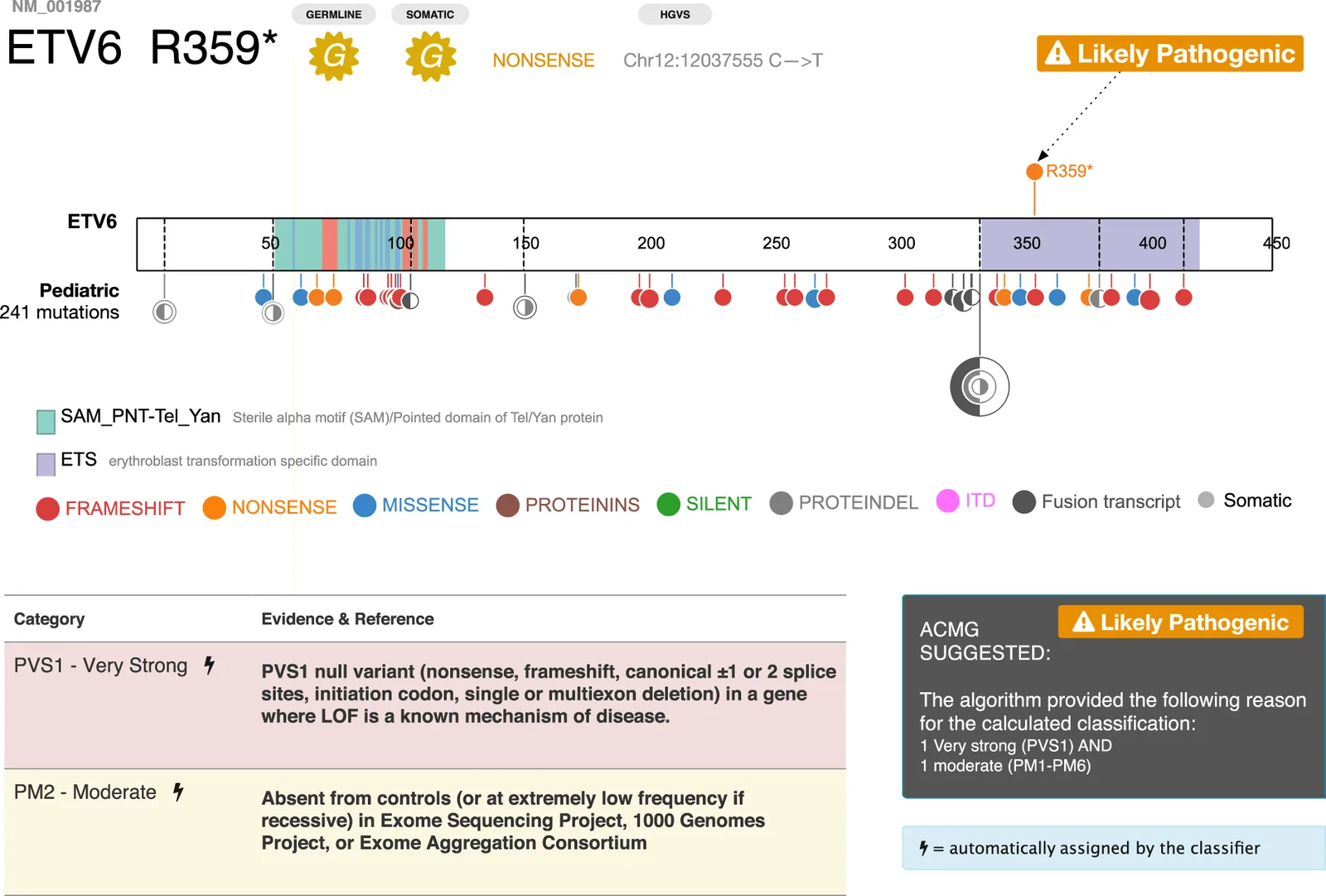
ACMG classification on *ETV6*. (*Top*) ProteinPaint display of somatic *ETV6* variants across 11 subtypes of pediatric leukemia, showing enrichment of loss-of-function mutations (frameshifts in red, nonsense variants in orange). Arrow indicates position of germline R359* variant. (*Bottom*) Detail of PeCanPIE ACMG classification interface for R359* variant.

A powerful feature of the variant detail page is an interactive graphical interface that allows a reviewer to enter a series of pathogenicity criteria evidence tags (e.g., population frequency, segregation, functional significance, and in silico prediction), along with supporting information such as PubMed IDs, to automatically calculate a five-tier classification: pathogenic (P), likely pathogenic (LP), variant of unknown significance (VUS), likely benign (LB), and benign (B) based on the ACMG algorithm. MedalCeremony can automatically generate ACMG classification tags for variants, which are prepopulated into PeCanPIE's classification interface. The following automatic tags are implemented per their ACMG specifications: PVS1 (truncating variant in a tumor suppressor or other loss-of-function gene), PM1 (somatic hotspot in COSMIC), PM2 (absent from ExAC non-TCGA or appearing at a frequency not greater than 0.0001) and the companion BA1 tag (>5% population frequency in ExAC non-TCGA), PM4 (in-frame protein insertions and deletions), PS1, and PM5 (amino acid comparisons made vs. pathogenic variants in ClinVar or those identified by the St. Jude germline variant review committee). Automatically assigned tags may be removed by the analyst if desired. This automation provides improved support over manual curation interfaces while still retaining analyst control over the ultimate classification decisions. As shown on the variant page for ETV6 Arg359Ter, the single gold medal variant detected in the patient with ALL was expert-classified as likely pathogenic because the mutation is present in a disease-related gene (i.e., *ETV6* is a pediatric ALL driver gene), is a loss-of-function null variant, and is not present in the ExAC non-TCGA database (Fig. 4).

Comparison of a germline variant with aggregated somatic variants can help inform germline classification for cancer predisposition genes. For example, family studies have identified a *PAX5* G183S germline mutation conferring susceptibility to B-ALL, which corresponds to somatic mutations detected in pediatric B-ALL and lymphoma (Shah et al. 2013). A similar profile was observed in the example WES data from an ALL patient presented in Figure 1B: MedalCeremony assigned a single gold medal—a novel *ETV6* nonsense variant within the ETS domain (NM_001987.4:c.1075C > T, NP_001978.1:p.Arg359Ter)—based on the criteria of truncation in a tumor suppressor gene. The ProteinPaint view embedded in the variant page confirmed that in *ETV6*, somatic mutations are dominated by loss-of-function mutations across pediatric leukemia (Fig. 4), consistent with the tumor suppressor gene model. This pathogenic variant was discovered in a research project, in which the MedalCeremony pipeline flagged this variant repeatedly in an affected family. Reviewers may enter custom evidence such as this into the interface for use during final classification.

**Figure 5.**
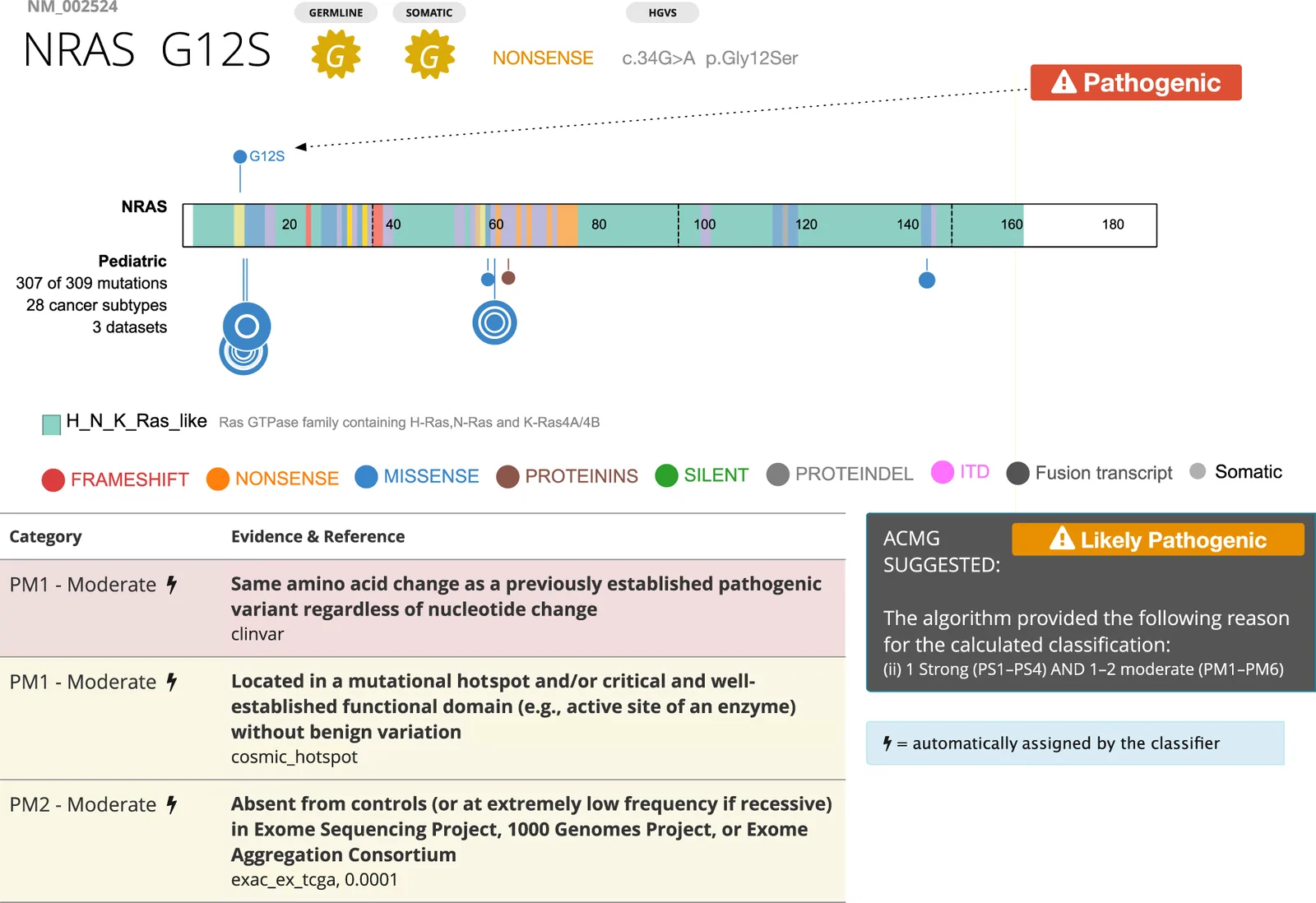
ACMG classification of NRAS G12S. (*Top*) ProteinPaint display of somatic NRAS variants across 28 cancer subtypes, showing hotspot at G12. Arrow indicates position of germline G12S variant. (*Bottom*) Detail of PeCanPIE ACMG classification interface for G12S variant. Automated classification detected a pathogenic ClinVar variant at the same amino acid position as well as a hotspot in COSMIC data.

Recurrent mutations, which include many gain-of-function variants, are reported either through matches to somatic mutation hotspots or to pathogenic variants in curated germline databases, such as ClinVar. Figure 5 shows a germline NRAS G12S variant detected in one patient to illustrate this case. Somatic data rendered in the ProteinPaint view shows a hotspot at G12, and automated classification detects pathogenic variations at the same amino acid position in ClinVar, as well as a hotspot in COSMIC data. This, coupled with the fact that biochemical assays have shown that this mutation would activate RAS (Schubbert et al. 2007), the final classification by the committee is pathogenic.

Collaborative features are also available: Users may invite others to access their results and work together on classification. This helps multidisciplinary researchers in disparate locales to perform distributed review and form their own variant classification committees if desired.

### Variant classification of noncancer genes

**Figure 6.**
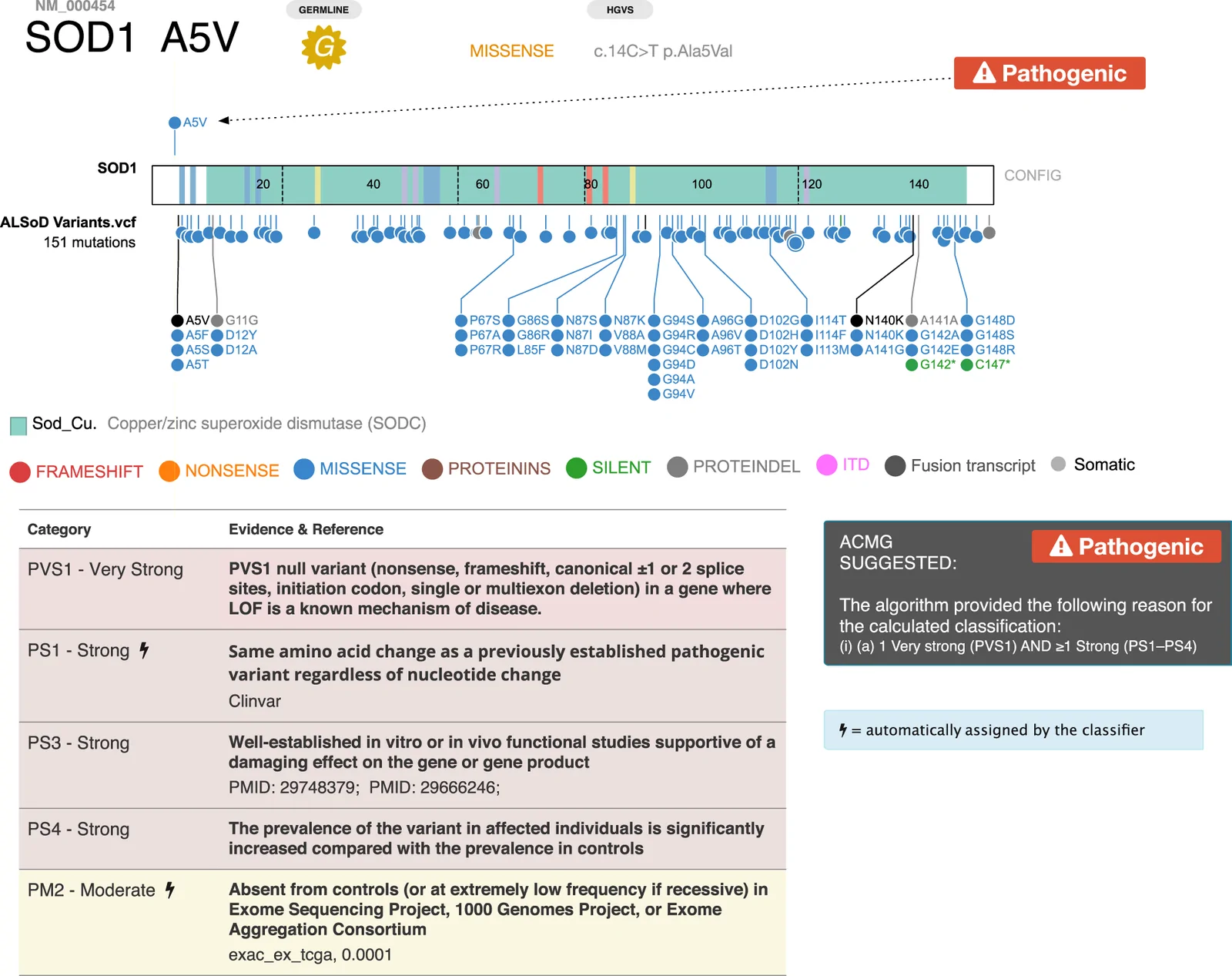
ACMG classification of SOD1 Ala5Val. (*Top*) ProteinPaint display of somatic SOD1 variants; arrow indicates position of Ala5Val. (*Bottom*) Detail of PeCanPIE ACMG classification interface for this variant.

Using the collaborative features described above, we are in the process of classifying and reviewing variants with the Clinical Research in ALS and Related Disorders for Therapeutic Development (CReATe) Consortium. To illustrate the utility of PeCanPIE to disease areas outside the realm of cancer genomics, we show the classification of a variant in the superoxide dismutase 1 (*SOD1*) gene reported by the ALS research community in the ALSoD database. Following ACMG classification criteria and taking into account the disease model as reported in OMIM, researchers at St. Jude are performing initial classifications, which undergo subsequent review by external researchers via secure remote login. As an example, we show a gold standard variant Ala5Val in the superoxide dismutase 1 (*SOD1*) gene (Fig. 6), located on Chromosome 21, which is the most common ALS-causing mutation in the United States (Rosen et al. 1994; Cudkowicz et al. 1997; Valentine and Hart 2003). Of note, the variant is displayed with reference to all variants in *SOD1* in the uploaded ALSoD data set, making it easy for researchers to determine the clustering of such variants for downstream targeted analyses. Using the semiautomated ACMG classification, we were able to determine that this variant is pathogenic as expected. Additional PubMed identifiers have been included in the ACMG PS3 criterion to allow external viewers to weigh the evidence accordingly.

### Use of PeCanPIE by the genetic research community

PeCanPIE was initially designed in support of large-scale germline variation analysis projects and was iteratively improved based on the feedback of an interdisciplinary group of researchers and individual users. Germline variants from the following studies have been analyzed thus far: (1) A study of germline variations in predisposition genes in 1120 children with cancer (Zhang et al. 2015) classified 890 variants, identifying 109 as pathogenic (P) and 25 as likely pathogenic (LP); (2) the St. Jude LIFE project, a follow-up study of 3006 long-term survivors of pediatric cancer (Wang et al. 2018), classified 3417 variants, including 188 P and 160 LP, for assessing genetic risk of secondary neoplasms in adulthood; and (3) the Genomes for Kids clinical research study of pediatric cancer patients (https://clinicaltrials.gov/ct2/show/NCT02530658), which is ongoing. The expert-curated decisions for the first two published studies were also reapplied to incoming variant classification requests.

We have also engaged the CReATe Consortium to expand PeCanPIE's application for noncancer studies. Variants in 522 ALS patients from CReATe are being curated for upload to PeCanPIE. A future direction by this working group is the inclusion of tracks displaying intrinsically disordered regions (IDRs), a hallmark of known ALS pathogenic genes, which will allow for further prioritization of novel variants.

In addition to large-scale genetic variation analysis, PeCanPIE also supports individual users who may have a small number of patient samples. Currently there are 228 registered individual users involved in a variety of human genetic studies (e.g., Mendelian diseases, adult and pediatric cancers) from >21 countries, who have collectively submitted >1100 jobs for analysis.

## Discussion

Although PeCanPIE's features partially overlap those of other available tools, we know of no other variant analysis system offering end-to-end processing with the same level of functionality. InterVar (Li and Wang 2017) provides some similar features, however its web-based implementation can analyze only a single variant at a time and only SNVs; it cannot handle indels, MNVs, or complex substitutions, nor can it batch-process variants. The offline version is more capable; however, it relies on the semicommercial ANNOVAR software (Wang et al. 2010), which requires paid licensing even for nonprofit organizations. CIViC (Griffith et al. 2017) offers a detailed curation model, but for predisposing variants it simply directs users to manually follow the ACMG classification guidelines. The ClinGen Pathogenicity Calculator (Patel et al. 2017) offers a comprehensive ACMG classification interface, but only works with a single variant at a time, lacks automation, and cannot batch-process variants. On the other end of the spectrum, CRAVAT (Masica et al. 2017) can batch-process collections of variants, but pathogenicity is ranked based on machine learning–based VEST and CHASM scores (Carter et al. 2009, 2013); in contrast, PeCanPIE provides more granular annotations and discrete ACMG-recommended evidence tags, which allow analysts to see and weigh these individual contributions to overall variant pathogenicity.

PeCanPIE also offers additional capabilities. Novel features include (1) tight integration of variant classification with the rich resource of somatic mutation data in pediatric cancer, which can be explored online via the embedded ProteinPaint view; (2) enhancement to splice variant annotation (Methods); (3) to aid analysts with literature review, PubMed references are extracted from ClinVar, VEP, COSMIC, and locus-specific databases and hyperlinked; (4) collaborative features allow users to invite others to share results or work together on classifying sets of variants; and (5) cloud-based implementation of PeCanPIE, which obviates the need for complex software installation and command-line workflows. This design also allows back-end analysis pipelines to be invoked independently from PeCanPIE for users who prefer direct or programmatic access over a graphical interface.

A limitation of the existing method is that precomputed damage-prediction algorithm scores are taken from the dbNSFP database, which only contains data for nonsilent SNVs. Although these annotations are unavailable for indels, because protein class annotations are taken into account by the scoring algorithm, high-impact events such as truncating variations will still be highly ranked. For variant population frequency filtering, we are currently using the non-TCGA subset of ExAC instead of gnomAD (Lek et al. 2016) because the gnomAD database contains TCGA samples; we plan to migrate to gnomAD once a TCGA-subtracted version becomes publicly available. Inaccuracy in public databases, such as misannotation of a rare germline variant as a somatic hotspot mutation, may lead to an over-rated gold medal assigned by MedalCeremony, which will require literature review for final pathogenicity classification.

In conclusion, the PeCanPIE platform significantly accelerates the variant classification process by automating many prerequisite steps, helping to prioritize potentially pathogenic variants in massively parallel sequencing data, and providing a robust platform for investigating variant pathogenicity in disease-related genes. Although PeCanPIE was developed and tested with pediatric cancer susceptibility as the initial focus, its use is now being expanded to other pediatric and adult diseases. Users are now able to specify custom gene lists to analyze appropriately to their diseases of interest, enabling disease-specific variant curation, and facilitating gene discovery. Its unique collaborative features allow scientists with different expertise to participate in the variant classification process, as demonstrated in the use of TP53 mutagenesis data generated from yeast and mammalian model organisms (Fig. 3D). PeCanPIE provides both clinical and basic science researchers alike an intuitive platform for evaluating alleles of interest in the context of existing data.

## Methods

### Disease-related gene lists

Gene lists are provided for cancer- and noncancer-related diseases (Supplemental Table S1). The cancer gene list was compiled from public resources and cancer genetic studies including (1) studies of germline mutations in predisposition genes in cancer patients (Zhang et al. 2015; Huang et al. 2018; Wang et al. 2018); (2) cancer predisposition genes compiled by Rahman (Rahman 2014); (3) the Cancer Gene Census (Futreal et al. 2004); and (4) driver genes identified in pediatric and adult pan-cancer studies (Gröbner et al. 2018; Ma et al. 2018). Publications were reviewed to confirm the presence of either loss-of-function or gain-of-function mutations in cancer driver genes, excluding those previously identified as having elevated mutation rates (e.g., *LRP1B*) (Lawrence et al. 2013) and those reported only as fusion partners. Other disease-related genes include cardiovascular, nonmalignant hematological, immunodeficiency, and amyotrophic lateral sclerosis (ALS)–related genes (Abel et al. 2013; Taylor et al. 2016) and genes from ACMG and Ambry Genetics incidental finding gene lists (Kalia et al. 2017). Filtering the input variants to disease-related genes helps focus on areas with relevant research interest and reduce the downstream processing burden, which is especially helpful for WGS data which may contain 4–5 million variants per sample. Users may choose to focus on one or more of these predefined disease categories for expert review or provide their own gene lists for custom analysis.

### Gene annotation and splice calling enhancement

Gene annotation is performed using the VEP pipeline (McLaren et al. 2016), which provides information on a variant basis for the affected gene and transcript, functional class (e.g., silent, missense, and nonsense), and effect on protein coding. We enhanced splice variant annotation by reclassifying silent or missense variants at exon boundaries, which may impact splicing (e.g., in *TP53*, NM_000546.5:c.375G > A, NP_000537.3:p.Thr125= and NM_000546.5:c.672G > A, NP_000537.3:p.Glu224=) (Supek et al. 2014; Soussi et al. 2017). Although not all of these variants will ultimately prove to be splice-related, these adjustments ensure additional scrutiny during expert review. A subsequent filtering step retains only variants in coding and splice-related regions. Silent variants are also kept because in rare cases they may cause aberrant splicing and thus be pathogenic. For example, ClinVar (Landrum et al. 2018) ID 90407 is a “silent” variant in the colon cancer predisposition gene *MLH1* (NM_000249.3:c.882C > T, NP_000240.1:p.Leu294=) that has been determined by an expert panel to be a pathogenic splice variant (Auclair et al. 2006). We refer to this enhanced pipeline as VEP+, which may also be run independently on St. Jude Cloud.

### Somatic medals

Although the PeCanPIE platform is intended to support only classification of germline variants, a basic somatic medal call is also provided to assist manual curation by considering the variant in a somatically acquired context. The somatic medal is primarily based on direct matches to somatic mutation hotspots or loss-of-function mutations in tumor suppressor genes annotated in the two somatic databases incorporated in this study, that is, PCGP and COSMIC. The separate logic can result in different medals assigned to somatic and germline. For example, the NOTCH1 R1350L variant in Figure 3 receives a bronze somatic medal and a germline silver medal; the more significant germline medal is attributable to a match in the ClinVar database, a resource that is not considered by the somatic classifier.

### St. Jude Cloud

Although PeCanPIE was designed as a web portal to maximize ease of use for non-bioinformaticians, two component pipelines are also publicly accessible. On its back-end, St. Jude Cloud (https://stjude.cloud) uses DNAnexus (https://www.dnanexus.com/), a platform in which user-created software pipelines can be installed and run on cloud computing instances. A DNAnexus account is required to use PeCanPIE for secure storage and to send notifications when submitted jobs are complete. Once a pipeline has been installed on DNAnexus, it is straightforward for nonexpert users to run it, either from a standardized web interface or a command-line client. We have installed two pipelines used by PeCanPIE on DNAnexus: VEP+ for variant annotation (app-stjude_vep_plus) and MedalCeremony for automated classification (app-stjude_medal_ceremony). These pipelines provide a wide variety of annotations which are then displayed by the PeCanPIE portal; bioinformaticians or others who would prefer direct access to the annotations can access them via the pipelines in a simple tab-delimited text format. The availability of these component pipelines on the cloud provides users and institutions straightforward, scalable access to the software, and our centralized maintenance allows all users to immediately benefit from updates and new features as they become available. An example workflow using the DNAnexus command-line client can be found in Supplemental Methods. For users who prefer not to use command-line tools, DNAnexus also provides a graphical interface for configuring and running the cloud pipelines. PeCanPIE is free for noncommercial use.

### Nomenclature standardization

We have observed that various variant databases that form the foundation of annotations for PeCanPIE vary in the structure and quality of variant specification. For example, databases may provide only protein-level annotations, only genomic annotations, or both. Likewise, there are many variations on the Human Genome Variation Society (HGVS)-like protein annotation nomenclature in circulation. The PeCanPIE code attempts to be flexible in parsing, standardizing, and formatting where possible; for example, protein annotations may use either three-character or one-character protein codes (e.g., “Ser” or “S”), and a number of variations on stop codon formatting have been observed (“Ter,” “Term,” “*,” “X,” and “Stop”). In some cases, partial information such as codon numbers were extracted from an otherwise incomplete annotation. Some databases also provide variations on the five-tier ACMG pathogenicity calls which PeCanPIE attempts to standardize into B/LB/VUS/LP/P for easier comparison. We believe these standardizations further improve the reliability of annotations and utility of information provided by the PeCanPIE platform.

### Example data

The ALL variants in Figure 1B were called from St. Jude sample SJNORM015857_G1 (Supplemental Methods) and uploaded to PeCanPIE. The Genome in a Bottle VCF used for Figure 1C is available from ftp://ftp-trace.ncbi.nlm.nih.gov/giab/ftp/release/NA12878_HG001/NISTv3.3.2/GRCh37/HG001_GRCh37_GIAB_highconf_CG-IllFB-IllGATKHC-Ion-10X-SOLID_CHROM1-X_v.3.3.2_highconf_PGandRTGphasetransfer.vcf.gz. This bgzip-compressed VCF file may be used directly with PeCanPIE.

### Software availability

PeCanPIE is available at https://platform.stjude.cloud/tools/pecan_pie and is one component of St. Jude Cloud (https://stjude.cloud/). Source code for the VEP+ and MedalCeremony pipelines and all scripts generated in this study are available as Supplemental Code and from https://github.com/mnedmonson/SJCRH/.

## Supplementary Material

Supplemental Material
