# Supplementary material for: Pediatric Cancer Variant Pathogenicity Information Exchange (PeCanPIE): a cloud-based platform for curating and classifying germline variants
Source: Genome Res. 2019 Sep;29(9):1555–65. doi: 10.1101/gr.250357.119 (PMC6724669; doi:10.1101/gr.250357.119)
Supplement: Supplemental Material [file supp_29_9_1555__index.html]

Pediatric Cancer Variant Pathogenicity Information Exchange (PeCanPIE): a cloud-based platform for curating and classifying germline variants — Supplemental Material 

# Pediatric Cancer Variant Pathogenicity Information Exchange (PeCanPIE): a cloud-based platform for curating and classifying germline variants

## Supplemental Material

- Supplemental\_Methods.pdf
- Supplemental\_Table\_S1.pdf
- Supplemental\_Code.zip
