# Supplementary material for: Pediatric Cancer Variant Pathogenicity Information Exchange (PeCanPIE): a cloud-based platform for curating and classifying germline variants
Source: Genome Res. 2019 Sep;29(9):1555–65. doi: 10.1101/gr.250357.119 (PMC6724669; doi:10.1101/gr.250357.119)
Supplement: Supplemental Material [file supp_gr.250357.119_Supplemental_Methods.pdf]

Supplemental Methods

Table of Contents

I. DNAnexus pipeline usage example
II. Bambino variant detection data
III. Source code

I. DNAnexus pipeline usage example

An example workflow using the DNAnexus command-line client follows:

1. Create a new DNAnexus account (https://www.dnanexus.com) and then log in at least once to PeCanPIE (https://pecan.stjude.cloud/pie/) with those credentials. This one-time login is required to grant your account permissions to run the cloud pipelines.
2. Install the DNAnexus toolkit and log into your account following the instructions in DNAnexus' quickstart guide (https://wiki.dnanexus.com/Command-Line-Client/Quickstart). This toolkit can be used to run any DNAnexus pipeline using the same command-line utilities. An optional token-based login method (using the "dx login --token" parameter) can be used to keep your account persistently logged in for a period of time of your choosing.
3. Upload the VCF file you want to analyze to the DNAnexus cloud: "dx upload my_vcf.vcf". This example assumes the variants are mapped to GRCh37; for a demo data file see the "Example Data" section in the main manuscript. Uploading a file will generate a unique DNAnexus file identifier (e.g. "file-FQgpk580FbXZ7zGz5y9gF1YV") which can be used in subsequent steps.
4. Run the VEP+ pipeline on the cloud-uploaded VCF: "dx run app-stjude_vep_plus -iinput_file=my_vcf.vcf -igenome_string=GRCh37-lite -igermline_reviewable_only=true". The input file name may use the uploaded filename if the name is unique in your project directory, or the unique file identifier generated by DNAnexus on upload. When complete, the pipeline will produce two output files in your cloud directory, "output_file" containing variant annotations, and "medal_prep_output_file" which is specially prepared as input for the MedalCeremony pipeline.

5. Run MedalCeremony on the “medal_prep_output_file” generated by the previous step: “dx run app-stjude_medal_ceremony -iinfile=medal_prep_output_file". The “output_file” produced by this process will contain the various annotations and medals used by PeCanPIE.

II. Bambino variant detection data

The ALL variants in Figure 1b were called from St. Jude sample SJNORM015857_G1. Variant calling was performed with Bambino using the “high 20” profile which consists of the following command-line parameters: “-min-quality 20 -min-flanking-quality 20 -min-alt-allele-count 3 -min-minor-frequency 0 -broad-min-quality 10 -mmf-max-hq-mismatches 4 -mmf-max-hq-mismatches-xt-u 10 -mmf-min-quality 15 -mmf-max-any-mismatches 6 -unique-filter-coverage 2 -no-strand-skew-filter”. The results were subsequently filtered to variants having a variant allele frequency of at least 20%, an average mapping quality of 20 for variant reads, at least 5 reads of coverage for the variant allele, bi-directional confirmation of the variant allele, and at least 20 reads of total coverage. The results were converted to VCF by an in-house script (“bambino2vcf.pl”, see “Source code” section below) and uploaded to PeCanPIE.

III. Source code

Source code for the VEP+ and Medal Ceremony pipelines and all scripts generated in this study is available in the Supplemental Code, and from https://github.com/mnedmonson/SJCRH/.
