# Supplementary material for: Pediatric Cancer Variant Pathogenicity Information Exchange (PeCanPIE): a cloud-based platform for curating and classifying germline variants
Source: Genome Res. 2019 Sep;29(9):1555–65. doi: 10.1101/gr.250357.119 (PMC6724669; doi:10.1101/gr.250357.119)
Supplement: Supplemental Material [file supp_gr.250357.119_Supplemental_Table_S1.pdf]

Table S1: disease-related genes

gene	Cancer	Cardiovascular	Immune	ALS	Hematological	ACMG Minimal Incidental	Ambry Expanded Incidental
AADACL4	**yes**						
ABCA4	**yes**						
ABCB7					**yes**		
ABCC9		**yes**					
ABCD1	**yes**						
ABI1	**yes**						
ABL1	**yes**						
ABL2	**yes**						
ACD	**yes**				**yes**		
ACKR3	**yes**						
ACSL3	**yes**						
ACSL6	**yes**						
ACTA2		**yes**				yes	
ACTB			**yes**				
ACTC1		**yes**				yes	
ACTN2		**yes**					
ACVR1	**yes**						
ACVR2A	**yes**						
ADA			**yes**				yes
AFF1	**yes**						
AFF3	**yes**						
AFF4	**yes**						
AICDA			**yes**				
AIP	**yes**						
AIRE			**yes**				yes
AK1					**yes**		
AK2			**yes**				
AKAP9	**yes**						
AKT1	**yes**						
AKT2	**yes**						
ALDH2	**yes**						
ALDOA					**yes**		
ALK	**yes**						yes
AMER1	**yes**						
ANK1					**yes**		
ANKRD1		**yes**					

ANKRD26					**yes**		
ANO6					**yes**		
AP3B1			**yes**				yes
APC	**yes**					yes	yes
APOB	**yes**	**yes**				yes	
APOL1			**yes**				
AR	**yes**						
ARHGAP26	**yes**						
ARHGEF12	**yes**						
ARID1A	**yes**						
ARID1B	**yes**						
ARID2	**yes**						
ARID5B	**yes**						
ARNT	**yes**						
ASPSCR1	**yes**						
ASXL1	**yes**						
ASXL2	**yes**						
ATF7IP	**yes**						
ATIC	**yes**						
ATL1				**yes**			
ATM	**yes**		**yes**				yes
ATP1A1	**yes**						
ATP2B3	**yes**						
ATR	**yes**				**yes**		
ATRX	**yes**						
AXIN1	**yes**						
AXIN2	**yes**						yes
B2M	**yes**						
BAG3		**yes**					
BAP1	**yes**						yes
BARD1	**yes**						
BAX	**yes**						
BAZ1A	**yes**						
BCL10	**yes**						
BCL11A	**yes**				**yes**		
BCL11B	**yes**						
BCL2	**yes**						
BCL2L11	**yes**				**yes**		
BCL3	**yes**						
BCL6	**yes**						
BCL7A	**yes**						

BCL9	**yes**						
BCL9L	**yes**						
BCOR	**yes**						
BCORL1	**yes**						
BCR	**yes**						
BIRC3	**yes**						
BLM	**yes**						yes
BLNK			**yes**				
BLOC1S3					**yes**		
BMP4	**yes**						
BMPR1A	**yes**						yes
BPGM					**yes**		
BRAF	**yes**						yes
BRCA1	**yes**					yes	yes
BRCA2	**yes**					yes	yes
BRD3	**yes**						
BRD4	**yes**						
BRIP1	**yes**						yes
BTG1	**yes**						
BTK	**yes**						yes
BUB1B	**yes**						yes
C11orf93	**yes**						
C15orf41					**yes**		
C16orf57					**yes**		
C17orf68	**yes**				**yes**		
C1QA			**yes**				
C1QB			**yes**				
C1QC			**yes**				
C1R			**yes**				
C1S			**yes**				
C2			**yes**				
C2orf44	**yes**						
C3			**yes**				
C4A			**yes**				
C4B			**yes**				
C4BPA			**yes**				
C4BPB			**yes**				
C5			**yes**				
C6			**yes**				
C7			**yes**				
C8A			**yes**				

C8B			**yes**				
C8G			**yes**				
C9			**yes**				
CACNA1D	**yes**						
CALR	**yes**						
CAMTA1	**yes**						
CANT1	**yes**						
CARD11	**yes**						
CARD9			**yes**				
CARS	**yes**						
CASC5	**yes**						
CASP10	**yes**						
CASP8	**yes**						
CASQ2		**yes**					
CAV3		**yes**					
CBFA2T3	**yes**						
CBFB	**yes**						
CBL	**yes**						yes
CBLB	**yes**						
CBLC	**yes**						
CCDC6	**yes**						
CCNB1IP1	**yes**						
CCND1	**yes**						
CCND2	**yes**						
CCND3	**yes**						
CCNE1	**yes**						
CD19			**yes**				
CD247			**yes**				
CD27			**yes**				
CD274	**yes**						
CD3D			**yes**				
CD3E			**yes**				
CD3G			**yes**				
CD40			**yes**				yes
CD40LG			**yes**				yes
CD46			**yes**				
CD55			**yes**				
CD59			**yes**				
CD74	**yes**						
CD79A	**yes**						
CD79B	**yes**						

CD81			**yes**				
CD8A			**yes**				
CDAN1					**yes**		
CDC25A	**yes**						
CDC73	**yes**						
CDH1	**yes**						yes
CDH11	**yes**						
CDK12	**yes**						
CDK4	**yes**						yes
CDK6	**yes**						
CDKN1A	**yes**						yes
CDKN1B	**yes**						yes
CDKN1C	**yes**						yes
CDKN2A	**yes**						yes
CDKN2C	**yes**						yes
CDX2	**yes**						
CEBPA	**yes**						yes
CEBPE			**yes**				
CFB			**yes**				
CFD			**yes**				
CFH			**yes**				
CFHR1			**yes**				
CFHR3			**yes**				
CFHR5			**yes**				
CFI			**yes**				
CFP			**yes**				
CHCHD7	**yes**						
CHD4	**yes**						
CHD7			**yes**				
CHEK2	**yes**						yes
CHIC2	**yes**						
CHMP2B				**yes**			
CHN1	**yes**						
CHRM2		**yes**					
CIC	**yes**						
CIITA	**yes**						
CLEC7A			**yes**				
CLP1	**yes**						
CLTC	**yes**						
CLTCL1	**yes**						
CNBP	**yes**						

CNOT3	**yes**						
CNTRL	**yes**						
COL1A1	**yes**						
COL2A1	**yes**						
COLEC11			**yes**				
CORO1A			**yes**				
COX6C	**yes**						
CR2			**yes**				
CREB1	**yes**						
CREB3L1	**yes**						
CREB3L2	**yes**						
CREBBP	**yes**						
CRLF2	**yes**						
CRTC1	**yes**						
CRTC3	**yes**						
CRYAB		**yes**					
CSF2RA			**yes**				
CSF3R					**yes**		
CSRP3		**yes**					
CTCF	**yes**						
CTNNB1	**yes**						yes
CTR9	**yes**						
CTSC			**yes**				
CUX1	**yes**						
CXCR4	**yes**		**yes**		**yes**		yes
CYBA			**yes**				
CYBB			**yes**				
CYCS					**yes**		
CYLD	**yes**						yes
CYP7B1				**yes**			
DAXX	**yes**						
DCLRE1C			**yes**				
DDB2	**yes**						yes
DDIT3	**yes**						
DDR2	**yes**						
DDX10	**yes**						
DDX3X	**yes**						
DDX41	**yes**						
DDX5	**yes**						
DDX6	**yes**						
DEK	**yes**						

DES		**yes**					
DGCR8	**yes**						
DHX15	**yes**						
DICER1	**yes**						yes
DIP2B	**yes**						
DIS3L2	**yes**						yes
DKC1	**yes**						yes
DMD		**yes**					
DNM2	**yes**						
DNMT3A	**yes**						
DNMT3B			**yes**				
DOCK8			**yes**				
DOLK		**yes**					
DROSHA	**yes**						
DSC2		**yes**				yes	
DSG2		**yes**				yes	
DSP		**yes**				yes	
DTNA		**yes**					
DTNBP1			**yes**				yes
DUSP10	**yes**						
DUX4L1	**yes**						
EBF1	**yes**						
ECT2L	**yes**						
EED					**yes**		
EGFR	**yes**						yes
EIF3H	**yes**						
EIF4A2	**yes**						
ELANE	**yes**						
ELF4	**yes**						
ELK4	**yes**						
ELL	**yes**						
ELN	**yes**						
EMD		**yes**					
EML4	**yes**						
EP300	**yes**						
EPAS1	**yes**				**yes**		yes
EPB41					**yes**		
EPB42					**yes**		
EPCAM	**yes**						yes
EPO	**yes**				**yes**		
EPOR					**yes**		

EPS15	**yes**						
ERBB2	**yes**						
ERBB3	**yes**						
ERBB4	**yes**						
ERCC1	**yes**						
ERCC2	**yes**						yes
ERCC3	**yes**						yes
ERCC4	**yes**						yes
ERCC5	**yes**						yes
ERCC6	**yes**						
ERCC8	**yes**						
ERG	**yes**						
ESR1	**yes**						
ETNK1	**yes**						
ETV1	**yes**						
ETV4	**yes**						
ETV5	**yes**						
ETV6	**yes**						
EWSR1	**yes**						
EXT1	**yes**						yes
EXT2	**yes**						yes
EZH2	**yes**						
EZR	**yes**						
FA2H				**yes**			
FADD			**yes**				
FAM175A	**yes**						
FAM46C	**yes**						
FANCA	**yes**						yes
FANCB	**yes**						
FANCC	**yes**						yes
FANCD2	**yes**						
FANCE	**yes**						yes
FANCF	**yes**						
FANCG	**yes**						yes
FANCI	**yes**						
FANCL	**yes**						
FANCM	**yes**						
FAP	**yes**						
FAS			**yes**				
FASLG			**yes**				
FAT1	**yes**						

FAT2	**yes**						
FAT4	**yes**						
FBN2	**yes**						
FBXO11	**yes**						
FBXO28	**yes**						
FBXW7	**yes**						
FCGR1A			**yes**				
FCGR2B	**yes**						
FCGR3A			**yes**				
FCN3			**yes**				
FCRL4	**yes**						
FERMT3			**yes**				
FEV	**yes**						
FGFR1	**yes**						
FGFR1OP	**yes**						
FGFR2	**yes**						yes
FGFR3	**yes**						yes
FGFR4	**yes**						
FH	**yes**						yes
FHIT	**yes**						yes
FHL2		**yes**					
FIP1L1	**yes**						
FLCN	**yes**						yes
FLG	**yes**						
FLI1	**yes**				**yes**		
FLNA	**yes**						
FLT3	**yes**						
FMR1	**yes**						
FNBP1	**yes**						
FOXA1	**yes**						
FOXL2	**yes**						
FOXN1			**yes**				
FOXO1	**yes**						
FOXO3	**yes**						
FOXO4	**yes**						
FOXP1	**yes**						
FOXP3			**yes**				
FPR1			**yes**				
FRG1	**yes**						
FRG2	**yes**						
FSTL3	**yes**						

FUBP1	**yes**						
FUS	**yes**						
G6PC			**yes**				
G6PC3	**yes**						
G6PD					**yes**		
GAR1	**yes**						
GAS7	**yes**						
GATA1	**yes**						
GATA2	**yes**						yes
GATA3	**yes**						
GATAD1		**yes**					
GBA2				**yes**			
GCLC					**yes**		
GFI1					**yes**		
GLA		**yes**				yes	
GMPS	**yes**						
GNA11	**yes**						
GNAQ	**yes**						
GNAS	**yes**						
GNB1	**yes**						
GOLGA5	**yes**						
GOPC	**yes**						
GP1BA					**yes**		
GP1BB					**yes**		
GP6					**yes**		
GP9					**yes**		
GPC3	**yes**						yes
GPHN	**yes**						
GPI					**yes**		
GPR101					**yes**		
GPX1					**yes**		
GREM1	**yes**						
GRIN2A	**yes**						
GRN				**yes**			
GSR					**yes**		
GSS					**yes**		
H3F3A	**yes**						
H3F3B	**yes**						
HABP2	**yes**						
HAX1	**yes**		**yes**		**yes**		yes
HBA1					**yes**		

HBB					**yes**		yes
HDAC2	**yes**						
HDAC7	**yes**						
HERPUD1	**yes**						
HEY1	**yes**						
HIP1	**yes**						
HIST1H3B	**yes**						
HIST1H4I	**yes**						
HK1					**yes**		
HLA-A	**yes**						
HLF	**yes**						
HMGA1	**yes**						
HMGA2	**yes**						
HMGN2P46	**yes**						
HNF1A	**yes**						yes
HNRNPA1				**yes**			
HNRNPA2B1	**yes**						
HOOK3	**yes**						
HOXA11	**yes**						
HOXA13	**yes**						
HOXA9	**yes**						
HOXB13	**yes**						yes
HOXC11	**yes**						
HOXC13	**yes**						
HOXD11	**yes**						
HOXD13	**yes**						
HPS1			**yes**				yes
HPS3			**yes**				yes
HPS4			**yes**				yes
HPS5			**yes**				yes
HPS6			**yes**				yes
HRAS	**yes**						yes
HSP90AA1	**yes**						
HSP90AB1	**yes**						
ICOS			**yes**				
ID3	**yes**						
IDH1	**yes**						yes
IDH2	**yes**						yes
IFNGR1			**yes**				
IFNGR2			**yes**				
IGF2R	**yes**						

IGH	**yes**						
IGHA1			**yes**				
IGHA2			**yes**				
IGHE			**yes**				
IGHG1			**yes**				
IGHG2			**yes**				
IGHG3			**yes**				
IGHG4			**yes**				
IGHM			**yes**				
IGK	**yes**						
IGKC			**yes**				
IGL	**yes**						
IGLL1			**yes**				
IKBKB	**yes**						
IKBKG			**yes**				
IKZF1	**yes**						
IKZF3	**yes**						
IL10RA			**yes**				
IL10RB			**yes**				
IL12B			**yes**				
IL12RB1			**yes**				
IL17F			**yes**				
IL17RA			**yes**				
IL2	**yes**						
IL21R	**yes**						
IL2RA			**yes**				
IL2RG			**yes**				yes
IL6ST	**yes**						
IL7R	**yes**						
ILK		**yes**					
IRAK4			**yes**				
IRF4	**yes**						
IRF8			**yes**				
IRS4	**yes**						
ITCH			**yes**				
ITGA2B					**yes**		
ITGB2			**yes**				
ITGB3					**yes**		
ITK	**yes**						
JAK1	**yes**						
JAK2	**yes**				**yes**		yes

JAK3	**yes**						
JAZF1	**yes**						
JMJD1C	**yes**						
JPH2		**yes**					
JUN	**yes**						
JUP		**yes**					
KAT6A	**yes**						
KAT6B	**yes**						
KBTBD4	**yes**						
KCNH2		**yes**				yes	
KCNJ5	**yes**						
KCNQ1		**yes**				yes	
KDM5A	**yes**						
KDM5C	**yes**						
KDM6A	**yes**						
KDR	**yes**						
KDSR	**yes**						
KEAP1	**yes**						
KIAA1549	**yes**						
KIF23					**yes**		
KIF5A				**yes**			
KIF5B	**yes**						
KIT	**yes**						yes
KLF1					**yes**		
KLF4	**yes**						
KLF6	**yes**						
KLK2	**yes**						
KMT2A	**yes**						
KMT2C	**yes**						
KMT2D	**yes**						
KRAS	**yes**						yes
KTN1	**yes**						
L1CAM				**yes**			
LAMA4		**yes**					
LAMA5	**yes**						
LAMP2		**yes**					
LASP1	**yes**						
LCK	**yes**						
LCP1	**yes**						
LDB3		**yes**					
LEF1	**yes**						

LHFP	**yes**						
LIFR	**yes**						
LIG1			**yes**				
LIG4			**yes**				
LMNA		**yes**				yes	yes
LMO1	**yes**						
LMO2	**yes**						
LPIN2			**yes**				
LPP	**yes**						
LRBA			**yes**				
LRIG3	**yes**						
LRRC8A			**yes**				
LYL1	**yes**						
LYST			**yes**		**yes**		yes
LZTR1	**yes**						
MAF	**yes**						
MAFB	**yes**						
MAGT1			**yes**				
MALAT1	**yes**						
MALT1	**yes**						
MAML2	**yes**						
MAP2K1	**yes**						
MAP2K2	**yes**						
MAP2K4	**yes**						
MAP3K1	**yes**						
MAP3K6					**yes**		
MAPK1	**yes**						
MAPT				**yes**			
MASP1			**yes**				
MASP2			**yes**				
MATR3				**yes**			
MAX	**yes**						yes
MBL2			**yes**				
MC1R	**yes**						
MCM4			**yes**				
MDM2	**yes**						
MDM4	**yes**						
MDS2	**yes**						
MECOM	**yes**						
MED12	**yes**						
MEFV			**yes**				

MEN1	**yes**					yes	yes
MET	**yes**						yes
MFN2	**yes**						
MGA	**yes**						
MITF	**yes**						yes
MKL1	**yes**						
MLF1	**yes**						
MLH1	**yes**					yes	yes
MLLT1	**yes**						
MLLT10	**yes**						
MLLT11	**yes**						
MLLT3	**yes**						
MLLT4	**yes**						
MLLT6	**yes**						
MLPH			**yes**				
MN1	**yes**						
MNX1	**yes**						
MPL	**yes**				**yes**		
MPO			**yes**				
MRE11A	**yes**						
MS4A1			**yes**				
MSH2	**yes**					yes	yes
MSH6	**yes**					yes	yes
MSI2	**yes**						
MSN	**yes**						
MTCP1	**yes**						
MTOR	**yes**						
MUC1	**yes**						
MURC		**yes**					
MUTYH	**yes**					yes	yes
MVK			**yes**				
MYB	**yes**						
MYBPC3		**yes**				yes	
MYC	**yes**						
MYCL	**yes**						
MYCN	**yes**						
MYD88	**yes**						
MYH11	**yes**					yes	
MYH6		**yes**					
MYH7		**yes**				yes	
MYH9					**yes**		yes

MYL2		**yes**				yes	
MYL3		**yes**				yes	
MYLK		**yes**				yes	
MYLK2		**yes**					
MYNN	**yes**						
MYO5A			**yes**				
MYOD1	**yes**						
MYOM1		**yes**					
MYOZ2		**yes**					
MYPN		**yes**					
NACA	**yes**						
NBN	**yes**						yes
NCF1			**yes**				
NCF2			**yes**				
NCF4			**yes**				
NCKIPSD	**yes**						
NCOA1	**yes**						
NCOA2	**yes**						
NCOA4	**yes**						
NCOR1	**yes**						
NCOR2	**yes**						
NDRG1	**yes**						
NEBL		**yes**					
NEXN		**yes**					
NF1	**yes**						yes
NF2	**yes**					yes	yes
NFE2L2	**yes**						
NFIB	**yes**						
NFKB2	**yes**						
NFKBIA			**yes**				
NFKBIE	**yes**						
NHEJ1			**yes**				
NHP2	**yes**						
NIN	**yes**						
NIPBL	**yes**						
NKX2-1	**yes**						
NLRC4					**yes**		
NLRP12			**yes**				
NLRP2					**yes**		
NLRP3			**yes**				
NOD2			**yes**				

NONO	**yes**						
NOP10	**yes**				**yes**		
NOTCH1	**yes**						
NOTCH2	**yes**						yes
NPAT	**yes**						
NPM1	**yes**						
NR4A3	**yes**						
NRAS	**yes**						yes
NSD1	**yes**						
NT5C2	**yes**						
NT5C3					**yes**		
NTHL1	**yes**						
NTRK1	**yes**						
NTRK3	**yes**						
NUMA1	**yes**						
NUP214	**yes**						
NUP98	**yes**						
NUTM1	**yes**						
NUTM2A	**yes**						
NUTM2B	**yes**						
OLIG2	**yes**						
OMD	**yes**						
OPTN				**yes**			
ORAI1			**yes**				
OTX2	**yes**						
P2RY12					**yes**		
P2RY8	**yes**						
PALB2	**yes**						yes
PALLD	**yes**						yes
PARN					**yes**		
PATZ1	**yes**						
PAX3	**yes**						
PAX5	**yes**						yes
PAX7	**yes**						
PAX8	**yes**						
PBRM1	**yes**						
PBX1	**yes**						
PCBP1	**yes**						
PCM1	**yes**						
PCSK7	**yes**						
PDCD1LG2	**yes**						

PDE4DIP	**yes**						
PDGFB	**yes**						
PDGFRA	**yes**						yes
PDGFRB	**yes**						
PDLIM3		**yes**					
PER1	**yes**						
PFKM					**yes**		
PFN1				**yes**			
PGK1					**yes**		
PHF6	**yes**						
PHOX2B	**yes**						yes
PICALM	**yes**						
PIGA			**yes**				
PIK3CA	**yes**						
PIK3CB	**yes**						
PIK3CD			**yes**				
PIK3R1	**yes**						
PIM1	**yes**						
PINK1	**yes**						
PKLR					**yes**		
PKP2		**yes**				yes	
PLAG1	**yes**						
PLCG1	**yes**						
PLCG2			**yes**				
PLN		**yes**					
PLP1				**yes**			
PLP2				**yes**			
PMEL	**yes**						
PML	**yes**						
PMS1	**yes**						
PMS2	**yes**					yes	yes
PNP			**yes**				
PNPLA6				**yes**			
POLD1	**yes**						yes
POLD3	**yes**						
POLE	**yes**						yes
POLH	**yes**						yes
POLQ	**yes**						
POT1	**yes**						
POU2AF1	**yes**						
POU5F1	**yes**						

PPARG	**yes**						
PPM1D	**yes**						
PPP2R1A	**yes**						
PPP2R5B					**yes**		
PPP2R5C					**yes**		
PPP2R5D					**yes**		
PPP6C	**yes**						
PRCC	**yes**						
PRDM1	**yes**						
PRDM16	**yes**						
PRDM9	**yes**						
PREX2	**yes**						
PRF1	**yes**						yes
PRKACA	**yes**						
PRKAG2		**yes**				yes	
PRKAR1A	**yes**						yes
PRKDC			**yes**				
PRRX1	**yes**						
PSIP1	**yes**						
PSTPIP1			**yes**				
PTCH1	**yes**						
PTCH2	**yes**						
PTCHD4	**yes**						
PTEN	**yes**					yes	yes
PTPN11	**yes**						
PTPN13	**yes**						
PTPRB	**yes**						
PTPRC			**yes**				
PTPRD	**yes**						
PTPRK	**yes**						
PTPRT	**yes**						
QKI	**yes**						
RAB27A					**yes**		
RABEP1	**yes**						
RAC1	**yes**						
RAC2					**yes**		
RAD21	**yes**						
RAD50	**yes**						
RAD51	**yes**						
RAD51B	**yes**						
RAD51C	**yes**						yes

RAD51D	**yes**						
RAF1	**yes**						
RAG1	**yes**						yes
RAG2	**yes**						yes
RALGDS	**yes**						
RANBP17	**yes**						
RANBP2	**yes**						
RAP1GDS1	**yes**						
RARA	**yes**						
RASA1					**yes**		
RASGRP2			**yes**				
RB1	**yes**					yes	yes
RBCK1			**yes**				
RBM10	**yes**						
RBM15	**yes**						
RBM20		**yes**					
RBM8A	**yes**						
RECQL	**yes**						
RECQL4	**yes**						yes
REEP1				**yes**			
REL	**yes**						
REST	**yes**						
RET	**yes**					yes	yes
RFX5			**yes**				
RFXANK			**yes**				
RFXAP			**yes**				
RHAG					**yes**		
RHBDF2	**yes**						yes
RHOA	**yes**						
RHOH	**yes**						
RHPN2	**yes**						
RIT1	**yes**						
RMI2	**yes**						
RNASEH2A			**yes**				
RNASEH2B			**yes**				
RNASEH2C			**yes**				
RNF168			**yes**				
RNF213	**yes**						
RNF43	**yes**						
ROS1	**yes**						
RPL10	**yes**						

RPL11	**yes**				**yes**		
RPL15	**yes**				**yes**		
RPL19	**yes**				**yes**		
RPL22	**yes**						
RPL26	**yes**				**yes**		
RPL35A	**yes**				**yes**		
RPL5	**yes**				**yes**		
RPN1	**yes**						
RPS10	**yes**				**yes**		
RPS14					**yes**		
RPS17	**yes**				**yes**		
RPS19	**yes**				**yes**		
RPS24	**yes**				**yes**		
RPS26	**yes**				**yes**		
RPS7	**yes**				**yes**		
RTEL1	**yes**						
RUNX1	**yes**						yes
RYR2		**yes**				yes	
SALL4	**yes**						
SAMD9	**yes**						
SAMD9L	**yes**						
SAMHD1			**yes**				
SBDS	**yes**						yes
SBF2					**yes**		
SCG5	**yes**						
SCN5A		**yes**				yes	
SCN9A	**yes**						
SDC4	**yes**						
SDHA	**yes**						yes
SDHAF2	**yes**					yes	yes
SDHB	**yes**					yes	yes
SDHC	**yes**					yes	yes
SDHD	**yes**					yes	yes
SEC23B					**yes**		
SELP	**yes**						
SEPT5	**yes**						
SEPT6	**yes**						
SEPT9	**yes**						
SERPING1			**yes**				
SET	**yes**						
SETBP1	**yes**						

SETD2	**yes**						
SF1					**yes**		
SF3A1					**yes**		
SF3B1	**yes**						
SFPQ	**yes**						
SGCD		**yes**					
SH2B3	**yes**						
SH2D1A	**yes**						
SH3GL1	**yes**						
SHOC2	**yes**						
SHROOM2	**yes**						
SI	**yes**						
SIRPA	**yes**						
SIX1	**yes**						
SIX2	**yes**						
SLC2A1					**yes**		
SLC34A2	**yes**						
SLC35C1			**yes**				
SLC37A4					**yes**		
SLC45A3	**yes**						
SLC46A1			**yes**				
SLC4A1					**yes**		
SLX4	**yes**						
SMAD2	**yes**						
SMAD3	**yes**					yes	
SMAD4	**yes**						yes
SMAD7	**yes**						
SMARCA4	**yes**						yes
SMARCB1	**yes**						
SMARCE1	**yes**						
SMC1A	**yes**						
SMC3	**yes**						
SMG8	**yes**						
SMO	**yes**						
SNX29	**yes**						
SOCS1	**yes**						
SOD1				**yes**			
SOS1	**yes**						yes
SOX2	**yes**						
SP110			**yes**				
SPAST				**yes**			

SPECC1	**yes**						
SPEN	**yes**						
SPG11				**yes**			
SPG7				**yes**			
SPINK5			**yes**				
SPOP	**yes**						
SPRED1	**yes**						yes
SPRTN	**yes**						
SPTA1					**yes**		
SPTB					**yes**		
SQSTM1				**yes**			yes
SRC	**yes**						
SRGAP3	**yes**						
SRP72					**yes**		
SRSF2	**yes**						
SRSF3	**yes**						
SS18	**yes**						
SS18L1	**yes**						
SSX1	**yes**						
SSX2	**yes**						
SSX4	**yes**						
STAG2	**yes**						
STAT1			**yes**				
STAT3	**yes**						yes
STAT5B			**yes**				
STAT6	**yes**						
STIM1			**yes**				
STK11	**yes**					yes	yes
STK4			**yes**				
STX11	**yes**						yes
STXBP2	**yes**						
SUFU	**yes**						yes
SUZ12	**yes**						
SYK	**yes**						
TAF15	**yes**						
TAL1	**yes**						
TAL2	**yes**						
TAP1			**yes**				
TAP2			**yes**				
TAPBP			**yes**				
TARBP2	**yes**						

TARDBP				**yes**			
TAZ					**yes**		
TBK1				**yes**			
TBL1XR1	**yes**						
TBR1	**yes**						
TBX1			**yes**				
TBX3	**yes**						
TBXA2R					**yes**		
TCAP		**yes**					
TCEA1	**yes**						
TCF12	**yes**						
TCF3	**yes**						
TCF7L1	**yes**						
TCF7L2	**yes**						
TCIRG1			**yes**				
TCL1A	**yes**						
TCL6	**yes**						
TCN2			**yes**				
TERC	**yes**				**yes**		
TERT	**yes**						yes
TET1	**yes**						
TET2	**yes**						
TFAP4	**yes**						
TFE3	**yes**						
TFG	**yes**						
TFPT	**yes**						
TFRC	**yes**						
TGFBR1	**yes**					yes	
TGFBR2	**yes**					yes	
THBD			**yes**				
THRAP3	**yes**						
TIAM1	**yes**						
TICAM1			**yes**				
TINF2	**yes**						
TLR3			**yes**				
TLX1	**yes**						
TLX3	**yes**						
TMC6			**yes**				
TMC8			**yes**				
TMEM127	**yes**						yes
TMEM43		**yes**				yes	

TMPRSS2	**yes**						
TNFAIP3	**yes**						
TNFRSF13C			**yes**				
TNFRSF14	**yes**						
TNFRSF17	**yes**						
TNFRSF1A			**yes**				
TNNC1		**yes**					
TNNI3		**yes**				yes	
TNNT2		**yes**				yes	
TOP1	**yes**						
TP53	**yes**					yes	yes
TP63	**yes**						
TPI1					**yes**		
TPM1		**yes**				yes	
TPM3	**yes**						
TPM4	**yes**						
TPR	**yes**						
TRA	**yes**						
TRAC			**yes**				
TRAF3			**yes**				
TRAF7	**yes**						
TRB	**yes**						
TRD	**yes**						
TRDN		**yes**					
TREX1			**yes**				
TRG	**yes**						
TRIM24	**yes**						
TRIM27	**yes**						
TRIM33	**yes**						
TRIP11	**yes**						
TRRAP	**yes**						
TSC1	**yes**					yes	yes
TSC2	**yes**					yes	yes
TSHR	**yes**						
TSPYL2	**yes**						
TTC37			**yes**				
TTL	**yes**						
TTN		**yes**					
TTR		**yes**					
TYK2	**yes**						
U2AF1	**yes**						

U2AF2					**yes**		
UBA2	**yes**						
UBQLN2				**yes**			
UBR5	**yes**						
UNC119			**yes**				
UNC13D	**yes**						yes
UNC93B1			**yes**				
UNG			**yes**				yes
UROD					**yes**		yes
USP6	**yes**						
USP7	**yes**						
USP8	**yes**						
USP9X	**yes**						
VCL		**yes**					
VCP				**yes**			
VHL	**yes**					yes	
VPREB1			**yes**				
VPS13B					**yes**		
VPS45					**yes**		
VTI1A	**yes**						
WAS	**yes**		**yes**		**yes**		yes
WDR64	**yes**						
WHSC1	**yes**						
WIF1	**yes**						
WIPF1					**yes**		
WRAP53	**yes**						
WRN	**yes**						yes
WT1	**yes**					yes	yes
WWTR1	**yes**						
XBP1	**yes**						
XK					**yes**		
XPA	**yes**						yes
XPC	**yes**						yes
XPO1	**yes**						
XPO5	**yes**						
XRCC2	**yes**						
YWHAE	**yes**						
ZAP70			**yes**				
ZBTB16	**yes**						
ZBTB24			**yes**				
ZBTB7A	**yes**						

ZEB2	**yes**						
ZFHX3	**yes**						
ZFP36L2	**yes**						
ZFPM1					**yes**		
ZIC1	**yes**						
ZMIZ1	**yes**						
ZMYM2	**yes**						
ZMYM3	**yes**						
ZNF217	**yes**						
ZNF331	**yes**						
ZNF384	**yes**						
ZNF521	**yes**						
ZRSR2	**yes**						
